# A biosensing system employing nanowell microelectrode arrays to record the intracellular potential of a single cardiomyocyte

**DOI:** 10.1038/s41378-022-00408-9

**Published:** 2022-06-27

**Authors:** Yuting Xiang, Haitao Liu, Wenjian Yang, Zhongyuan Xu, Yue Wu, Zhaojian Tang, Zhijing Zhu, Zhiyong Zeng, Depeng Wang, Tianxing Wang, Ning Hu, Diming Zhang

**Affiliations:** 1 Department of Obstetrics and Gynecology, Affiliated Dongguan People’s Hospital, Southern Medical University, Dongguan, 523058 China; 2 Research Center for Intelligent Sensing Systems, Zhejiang Laboratory, Hangzhou, 311100 China; 3 Research Center for Humanoid Sensing, Zhejiang Laboratory, Hangzhou, 311100 China; 4 Key Laboratory of Novel Target and Drug Study for Neural Repair of Zhejiang Province, School of Medicine, School of Computer & Computing Science, Zhejiang University City College, Hangzhou, 310015 China; 5 School of Brain Science and Brain Medicine, Zhejiang University, Hangzhou, 310058 China; 6 School of Automation, Nanjing University of Science and Technology, Nanjing, 210094 China; 7 College of Energy and Power Engineering, Nanjing University of Aeronautics and Astronautics, Nanjing, 210016 China; 8 E-LinkCare Meditech Co., Ltd, Hangzhou, 310011 China; 9 ZJU-Hangzhou Global Scientific and Technological Innovation Center, Department of Chemistry, Zhejiang University, Hangzhou, 310058 China

**Keywords:** Bionanoelectronics, Sensors

## Abstract

Electrophysiological recording is a widely used method to investigate cardiovascular pathology, pharmacology and developmental biology. Microelectrode arrays record the electrical potential of cells in a minimally invasive and high-throughput way. However, commonly used microelectrode arrays primarily employ planar microelectrodes and cannot work in applications that require a recording of the intracellular action potential of a single cell. In this study, we proposed a novel measuring method that is able to record the intracellular action potential of a single cardiomyocyte by using a nanowell patterned microelectrode array (NWMEA). The NWMEA consists of five nanoscale wells at the center of each circular planar microelectrode. Biphasic pulse electroporation was applied to the NWMEA to penetrate the cardiomyocyte membrane, and the intracellular action potential was continuously recorded. The intracellular potential recording of cardiomyocytes by the NWMEA measured a potential signal with a higher quality (213.76 ± 25.85%), reduced noise root-mean-square (~33%), and higher signal-to-noise ratio (254.36 ± 12.61%) when compared to those of the extracellular recording. Compared to previously reported nanopillar microelectrodes, the NWMEA could ensure single cell electroporation and acquire high-quality action potential of cardiomyocytes with reduced fabrication processes. This NWMEA-based biosensing system is a promising tool to record the intracellular action potential of a single cell to broaden the usage of microelectrode arrays in electrophysiological investigation.

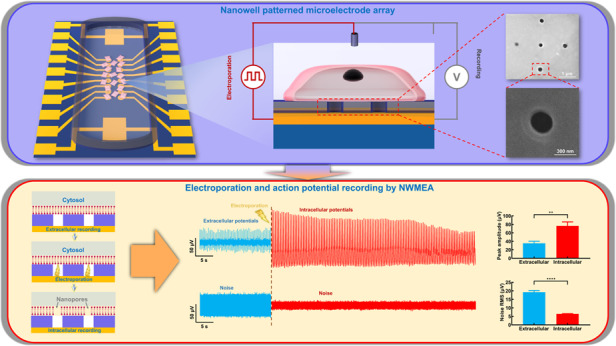

## Introduction

Cardiovascular disease (CVD) is the leading cause of death, accounting for ~32% of total mortality worldwide^1,2^. It is estimated that annual cases of unexpected sudden cardiac death (SCD) exceed 180,000 in the USA^3^ and 500,000 in China^4^. The serious mortality of CVD has incited numerous drug studies for treatment purposes, and cardiotoxicity is one of most important adverse effects assessed in both novel drug development and postmarketing drug surveillance^5–7^. In fact, approximately 45% of drug withdrawals from the market account for their potential cardiotoxicity^8,9^. Hence, assessing the cardiotoxicity of drug candidates during the drug development process is essential to reduce the withdrawal risk of new drugs.

Electrophysiological recording has been a widely used method to evaluate drug cardiotoxicity in vivo and in vitro, and various cardiac electrophysiological tools have been developed to assess drug cardiotoxicity over a broad scope, ranging from subcellular-level tests to large-scale animal tests^10–12^. Cardiovascular electrophysiological techniques employing animal models, such as dogs, monkeys, and mice, can contribute as an in vivo screening tool to evaluate drug-induced cardiovascular disorders in clinical trials^13,14^. However, their preclinical applications to screen a large library of drug candidates in a high-throughput way are restricted by the high cost of animal experiments. To overcome these limitations, cell-based electrophysiological techniques have emerged with in vitro cultured cardiomyocytes as physiologically relevant models for cardiotoxicity assessment^15–17^. As cardiomyocytes inherit electrophysiological properties from animal hearts, cell-based electrophysiological techniques can be utilized for efficient testing of drug cardiotoxicity and thereby can significantly reduce the cost of animal experiments. Apparently, cell-based techniques have provided a good choice to balance the cost and throughput in large-scale drug screening.

Patch clamp and microelectrode array (MEA) are two commonly used techniques for cell-based cardiac electrophysiological measurement. In the patch clamp technique, the micropipette tip is brought in contact with a patch of the cell membrane, which is destroyed by suction to record the intracellular voltage or current^18–21^. The sealing after the destruction of the local cell membrane gives good electrical coupling between the electrode and clamped cell, enabling effective recordings of intracellular signals with patch clamp. However, patch clamp has two limitations. First, it can only provide an effective recording within a limited duration from several minutes to hours due to irreversible damage to the cell membrane. Second, it sets a high bar for users because precise operations are required in the control of micropipettes to touch and break the cell membrane, which is time-consuming. Planar MEA can achieve efficient cardiac electrophysiology recording in a noninvasive, long-term recording and high-throughput way^22–24^. However, due to the planar geometry of the electrode, MEA can only record extracellular potentials with a low signal-to-noise ratio (SNR). In addition, the micron-sized electrode of MEA always records the electrical potentials of the cell population rather than a single cell. Planar MEAs are not qualified in some applications that require intracellular potential measurements of single cells, such as cardiotoxicity assessments based on electrical recordings of individual cardiomyocytes.

Owing to recent advances in micro- and nanofabrication, three-dimensional nanostructures, such as nanopillars and nanovolcanos, have been fabricated on microelectrode surfaces^25,26^. These nanostructures can penetrate the cell membrane so that the intracellular potentials are available to the MEA electrodes with improved cell-electrode coupling, which enables the MEA technique to record the high-quality intracellular potential of cells. Although these works have demonstrated that the nanostructured MEA can obtain electrophysiological recordings with higher intracellular SNR compared to the planar MEA, the nanostructures on the MEA always grow to several microns in size to ensure good engulfing of nanostructures into the cell membrane and thus can induce a certain amount of irreversible and continuous damage to cells in the electroporation processes. Moreover, the upward growth of nanostructures is both time- and money-consuming, which has limited the large-scale production and application of nanostructured MEAs in intracellular recording^27–29^. To date, it is still an ongoing challenge for researchers to fabricate a low-cost nanostructured MEA that can measure the high-quality intracellular potential of a single cell with controllable and minimal damage.

**Fig. 1:**
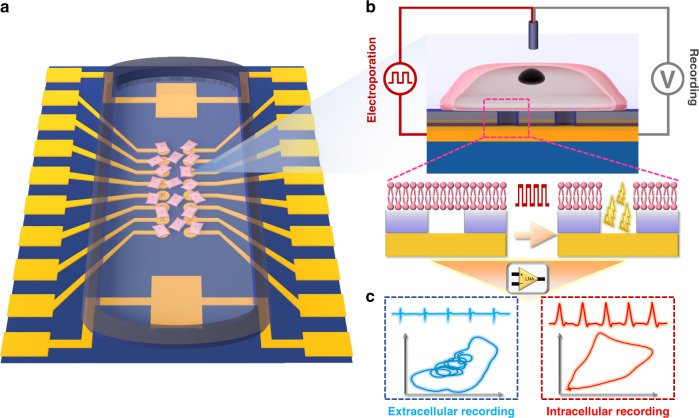
Schematic of the biosensing system employing NWMEA for electrophysiological recording. Schematic of the biosensing system employing NWMEA for electrophysiological recording. **a** Cardiomyocytes were cultured on the 16-channel NWMEA device for intracellular potential recording. **b** NWMEA penetrated the cardiomyocyte membrane by electroporation and recorded intracellular action potentials. **c** The nonlinear dynamic analysis transforming time-domain signals into phase domain signals worked together with profile comparison to provide a comprehensive understanding of NWMEA recording

In this work, we presented a nanowell microelectrode array (NWMEA)-based biosensing system. The system can overcome the aforementioned limitations of conventional MEA systems and is able to record intracellular potential from a single cardiomyocyte. Specifically, the NWMEA was first fabricated by drilling five nanowells at the center of each electrode of the conventional planar MEA. The electroporation applied in the NWMEA can generate electric fields across the cardiomyocytes and then penetrate the cardiomyocyte membrane to form nanopores. Nanopore penetration helped the microelectrodes and cardiomyocytes form good electrical coupling between each other for intracellular potential recording. The micron-sized nanowell array on the microelectrodes enabled the penetration point to stay under a single cardiomyocyte for permeability and potential recording. To demonstrate the performance of the system, we recorded the extra- and intracellular potential of a single cardiomyocyte before and after electroporation, respectively, and successfully captured the intracellular potential, whose SNR was approximately 4 times that of the extracellular potential. We also performed time-domain signal analysis and nonlinear dynamic analysis to further compare the signals recorded by the NWMEA and those recorded by nanopillar microelectrode arrays (NPMEAs). Although the NWMEA recorded a relatively lower action potential amplitude than the NPMEA, the NWMEA is able to record qualified signals with reduced fabrication processes. Overall, the biosensing system integrated with NWMEA provided a powerful tool to probe and analyze intracellular potential for electrophysiological study. A schematic of the biosensing system with the NWMEA is described in Fig. 1.

## Experimental methods

### Au microelectrodes and nanowell array fabrication

There are two steps in the fabrication of NWMEAs: fabrication of the planar microelectrode and integration of the nanowell array. Both steps were completed by standard lift-off and FIB processes. The planar microelectrode array was first fabricated with conventional microfabrication techniques, including photolithography, deposition, and lift-off processes. First, a 4-inch silicon wafer (University Wafer, USA) was cleaned with piranha solution (H_2_O_2_ & H_2_SO_4_, Sigma-Aldrich, USA) to work as an insulated substrate for microelectrode fabrication. The microelectrodes and leads were designed in AutoCAD software (Autodesk Inc., USA) with a 20 mm × 20 mm region for each piece. Through the lift-off process and metallization according to the predesigned photomask pattern, a 10-nm-thick Cr layer and a 50-nm-thick Au layer were successively deposited on the wafer as the microelectrodes and conductive lines, respectively. Finally, a 200-nm-thick SiO_2_ passivated layer was deposited on the microelectrodes and conductive lines for electrical insulation by radio frequency (RF) magnetron sputtering.

After the fabrication of the planar microelectrode array, nanowell arrays were fabricated on the microelectrodes by a focused ion beam (FIB) with Lyra3 FEG-SEM/FIB equipment (Tescan, Czech). First, a 5-nm-thick Ni layer was deposited on the passivated layer as a protective layer for FIB processing. Second, scanning electron microscopy (SEM) (Tescan VEGA3, Czech) was used to image the microelectrodes and locate the center of the microelectrodes. The Ga^+^ FIB hit the center of the microelectrodes to etch a nanowell, which passed through the passivated layer to access the Au layer of microelectrodes. The other four nanowells were milled around the central nanowell by the FIB as well, providing a nanowell array for efficient electroporation. Since wells with small sizes for electroporation can facilitate low-voltage discharge on the cell membrane, nanosized wells were preferred in our work. However, the nanowell size should be large enough to ensure contact impedance between the cell and electrode. Hence, to take these two facts into account, a tradeoff was made. In addition, the nanowell size should be on the same order of magnitude as the nanopores created by electroporation, which can prevent the loss of potential signals. Considering the previously reported nanowell size (on the scale of hundreds of nanometers) applied in electroporation-based cell transfection^30^, we chose a nanowell size of ~300 nm in our work. The distances between each nanowell were ~1 µm. Finally, the NWMEA with locally exposed microelectrodes on a round pad was obtained by etching the remainder of the Ni protective layer with TFG nickel etchant (Transene, USA). Sixteen round pads with diameters of 30 μm were arranged in two parallel lines as detecting electrodes, while two large rectangular pads with a size of 3 × 2 mm were placed on either side of the detecting electrode region as reference electrodes. All electrodes were connected to the large pads on the edge of the substrate by 20-μm-thick lines for device packaging. A customized polydimethylsiloxane (PDMS) chamber was assembled on each piece of the NWMEA to form a biodevice for the cell culture.

### HL-1 rat cardiomyocyte culture

The HL-1 rat cardiomyocytes were cultured on the NWMEA biodevice to test the cellular electroporation and record electrical potential. In the preparation before cell culture, the NWMEA biodevice was incubated overnight with 5 µg/mL fibronectin in 0.02% gelatin solution at 4 °C to enhance cell attachment on the device surface. During the cell culture, the frozen HL-1 cardiac muscle cell line (Louisiana State University Health Science Center, USA) was thawed in a 37 °C water bath and incubated in an HL-1 expansion medium to recover the cardiomyocytes. To purify the HL-1 cardiomyocytes, they were pelleted by centrifuging the HL-1 expansion medium including the cells at 300 × *g* for 2–3 min and resuspended into 10 to 15 mL of fresh HL-1 expansion medium. The HL-1 cardiomyocyte suspension was transferred into the NWMEA biodevice at a density of 10^4^ cells/cm^2^ and maintained in a standard incubator at 37 °C and 5% CO_2_ for the subsequent experiments. The HL-1 expansion medium was made from a mixture of 43.5 mL Claycomb basal medium (51800 C, Sigma-Aldrich), 5 mL qualified FBS (TMS-016-B, EMD Millipore), 0.5 mL l-glutamine at 200 mM (A0937, Sigma-Aldrich), 0.5 mL norepinephrine at 10 mM (TMS-002-C, EMD Millipore) and 0.5 mL penicillin/streptomycin at 100X (TMS-AB2-C, EMD Millipore). The cardiomyocyte culture was imaged by an upright widefield microscope (Olympus BX63, Japan).

### Immunofluorescence staining

The HL-1 cardiomyocytes were rinsed with phosphate-buffered saline (PBS, 806552, Sigma-Aldrich) and fixed in 4% paraformaldehyde (P6148, Sigma-Aldrich) at room temperature for 30 min. After washing three times with prechilled PBS, the HL-1 cardiomyocytes were permeabilized with 0.15% Triton X-100 (T8787, Sigma–Aldrich) in PBS for 15 min and blocked with 1% bovine serum albumin (BSA, A1933, Sigma–Aldrich) for 30 min at room temperature. For cytoplasmic labeling, HL-1 cardiomyocytes were incubated with a primary antibody against α-actin (A3853, Sigma-Aldrich) at a dilution of 1:1000 at 4 °C overnight and washed with PBS three times to remove the remaining antibodies. Then, the cardiomyocytes were incubated with Cy3-labeled secondary goat anti-mouse IgG antibodies (AP130C, Sigma-Aldrich) for 2 h at room temperature without light. The cardiomyocytes were washed with PBS three times again to remove the unbound fluorescent probes. For nuclear labeling, the cardiomyocytes were incubated with 4′,6-diamidino-2-phenylindole (DAPI, D9542, Sigma-Aldrich) for 10 min at room temperature and washed with PBS to remove the unbound DAPI. Fluorescence images were captured by a laser confocal fluorescence microscope (Nikon A1HD25, Japan).

### Electrical impedance spectroscopy scanning

To explore the electrochemical properties of the NWMEA biodevice, the electrical impedance spectra of the biodevice filled with Claycomb culture medium (51800C, Sigma) were measured on an electrochemical workstation (Reference 600+, Gamry Instruments). The three-electrode system consisted of an external Ag/AgCl electrode as a reference electrode, a Au pad as a counter electrode, and a nanowell microelectrode as a working electrode. The scanning frequency of the measurement was from 10 Hz to 10^6^ Hz, and the excitation signal was 20 mV in amplitude.

### Electrophysiological measurement and electroporation

Electrophysiological recording of the HL-1 cardiomyocytes was performed by a 16-channel voltage amplifier system (USB-ME16-FAI, Multichannel Systems). The faint original signals were amplified 1000 times through a special preamplifier of the system and eventually sampled at 25 kHz by specialized software on the computer. Electroporation of the HL-1 cardiomyocytes was performed by a waveform generator (DG 1032Z, Rigol). A train with 3 Vp-p amplitude and 200 µs biphasic pulses was applied on the NWMEA that served as the positive electrodes for electroporation, while the Au pad on the NWMEA biodevice served as the negative electrode for electroporation. The electrophysiological measurement and cellular electroporation were both performed on the NWMEA biodevice with 16 simultaneous channels. The temperature of the incubator was kept at 37 °C during the electrophysiological measurement and cellular electroporation.

### Signal processing and statistical analysis

Signal processing and analysis were performed with a customized LabVIEW program. The recordings of extra and intracellular potentials in this work were nonstationary signals in the time series. Hence, digital wavelet transforms (DWTs) were used to detrend and denoise the signals. Peaks of the cellular potentials were detected by multiresolution wavelet analysis^31^. In the statistical analysis of the signal dynamic change from NWMEA electroporation recording, mean values of peak amplitude, noise RMS and SNR were calculated every 2 s and averaged for 6 channels. To compare the performance of NWMEA and NPMEA electroporation in the phase domain, the recorded signals at selected time points (0, 2, 5, 10, 20 min) were reconstructed in two-dimensional phase space and averaged over 20 s after the selected time points. The shape index was introduced to compare the averaged graphs after electroporation with those before electroporation and those at the moment of electroporation. The shape index was calculated using the matchShapes function of the OpenCV library based on the Hu moment. The shape indices from different time points were averaged at the selected time points for 6 channels. Statistical analyses were performed in GraphPad Prism 8.0. All results are presented as the mean ± standard error of the mean (SEM), and groups were compared by unpaired *t* test.

### Nonlinear dynamic analysis of extra and intracellular potential signals

To study the complex dynamic properties of the electrophysiological signals, nonlinear dynamic analysis was performed to analyze the extracellular and intracellular potential signals in the current work. The extracellular and intracellular potential signals were represented by the vector:

$${{{\boldsymbol{x}}}}({{{\boldsymbol{i}}}}) = \left\{ {x_1,x_2,x_3, \cdots ,x_N} \right\}$$

where N is the number of points in the time series. Nonlinear dynamic parameters, including delay time *τ* and embedding dimension *m*, were employed to transform the extracellular and intracellular potential signals into two-dimensional phase space^32,33^. The reconstructed signals in phase space were

$${{{\boldsymbol{X}}}}({{{\boldsymbol{i}}}}) = \left\{ {x_i,x_{i + \tau },x_{i + 2\tau }, \cdots ,x_{i + \left( {m - 1} \right)\tau }} \right\}$$

where *τ* is the delay time and *m* is the embedding dimension. The delay time *τ* represents the delays between consecutive samples in the reconstructed phase space, which can be calculated by the autocorrelation function:

$$R_{xx}\left( u \right) = \frac{1}{N}\mathop {\sum }\limits_{i = 0}^{N - 1} x\left( i \right)x\left( {i + u} \right)$$

where *R*_*xx*_ **(***u***)** is the autocorrelation coefficient of the digital series {*x***(***i***)**, *i* = 0, 1, 2, …, *N***}**. When *R*_*xx*_ (*u*) dropped to $1 - \frac{1}{e}$ of its original value *R*_*xx*_ (0), the corresponding value of *u* was set as the optimal time delay *τ*. The embedding dimension *m* was set as 2 in the current study. Hence, the time series reconstructed in the phase space can be written as follows:

$$\left\{ {X\left( i \right),1 \le i \le N - 1} \right\},\;{\rm{and}}\;X\left( i \right) = \left\{ {x\left( i \right),\;x\left( {i + \tau } \right)} \right\}$$

*x*(*i*) and *x*(*i* + *τ*) were used as the *X* axis and *Y* axis to plot the two-dimensional phase space reconstruction, respectively.

## Results

### Fabrication and characterization of the NWMEA

**Fig. 2:**
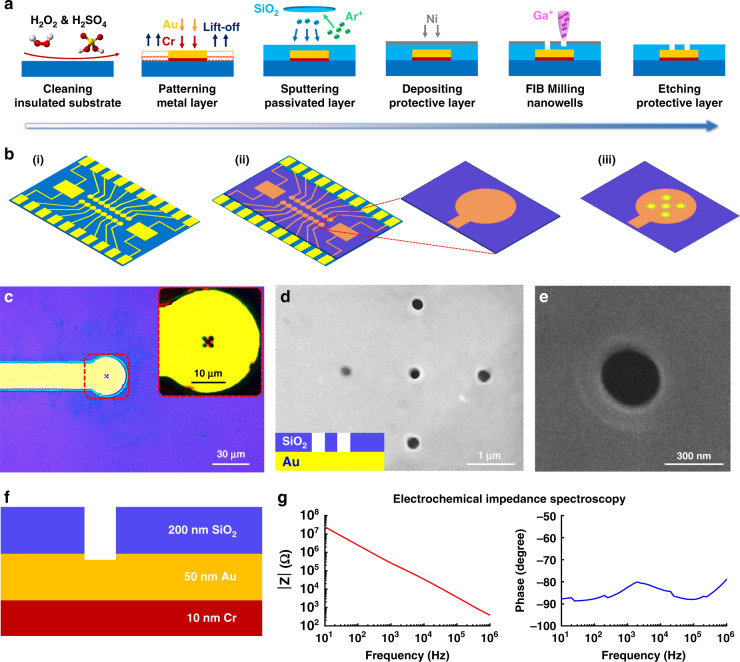
Fabrication and characterization of NWMEA. **a** The microfabrication process flow for the NWMEA. **b** Schematic view of the NWMEA that incorporated nanowells. **c** Optical imaging of a single electrode of the NWMEA. The inset inside the red dotted box shows enlarged details of the electrode. **d** SEM image of the nanowell array on the microelectrode. The inset shows the material and structure of the nanowell array. **e** SEM image of a single nanowell with a diameter of ~300 nm. **f** Schematic of the single nanowell from top to bottom to show the electrical conductivity of the NWMEA. **g** Electrochemical impedance spectroscopy of the NWMEA. The left and right panels show the impedance and phase changes with frequency, respectively

To penetrate and probe single cardiomyocytes, NWMEAs were fabricated by integrating nanowell arrays on the microelectrode of the MEA device (Fig. 2a, b, see “Methods”). The individual microelectrode of the NWMEA was visualized by using optical microscopy imaging (Fig. 2c). The imaging showed that the circular microelectrode was ~30 μm in diameter and that the nanowell array was located in its center. SEM was employed to further visualize the structure and size of the nanowell array (Fig. 2d, e). SEM showed that one of the five nanowells was located in the center of the array and that the other four nanowells were distributed at equal distances from the central nanowell (~1 μm). Imaging of the individual nanowells showed that the nanowells were ~300 nm in diameter (Fig. 2e). The images above have demonstrated success in the fabrication of the nanowell array. Considering that cardiomyocytes were always approximately several tens of microns in size, the nanowell arrays on the electrodes were fabricated to be much smaller than individual cardiomyocytes and enabled single cell electroporation and potential recording.

We further performed electrochemical impedance spectroscopy to check whether the nanowells accessed the conductive Au layer of the microelectrode as designed in fabrication (Fig. 2f). The access of nanowells to the conductive layer electrically coupled cells and microelectrodes, which was crucial for cellular electroporation and electrophysiological recording. As shown in Fig. 2g, the impedance modulus decreased with frequency and was approximately 270 kΩ at 1 kHz. The impedance phase fluctuated around -90 degrees with frequency. The impedance modulus of the NWMEA was larger than that of some reported MEAs due to the extremely small unpassivated surface area of NWMEA electrodes of approximately 0.35 μm^2^ compared to that of microelectrodes of approximately several hundreds of square microns^34^. The large impedance modulus could decrease the current passing through the cell-electrode interface during cell electroporation. However, the small area in turn increased the current density through the electrodes and cell membrane, which compensated for the decreasing current induced by the large impedance and maintained the efficiency of cell electroporation. The electrical characteristics of the NWMEA are similar to those of capacitive electrodes; these characteristics enable scientists to perform good cellular electroporation and electrophysiological recording^34–36^.

### Extracellular and intracellular potential recording by the NWMEA

**Fig. 3:**
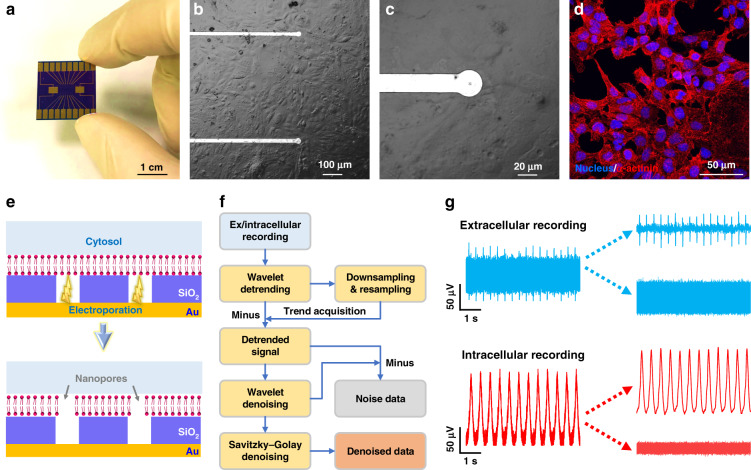
Culture and electroporation of cardiomyocytes for extracellular and intracellular potential recording using NWMEA. Culture and electroporation of cardiomyocytes for extracellular and intracellular potential recording using NWMEA. **a** Photo of the NWMEA device assembled on an adaptive printed circuit board. **b** Optical microscope imaging of HL-1 cardiomyocytes cultured on the NWMEA biodevice. **c** Optical microscope imaging of a single electrode of the NWMEA biodevice when covered by a cardiomyocyte. **d** Immunostaining image (merged) of HL-1 cardiomyocytes stained with DAPI and Cy3-labeled antibody. The DAPI- and Cy3-labeled nuclei of the cardiomyocytes in blue fluorescence and α-actinin of the cardiomyocytes in red fluorescence. **e** Schematic drawing showing that electroporation perforated the cardiomyocyte membrane to achieve intracellular potential recording on the NWMEA device. **f** Workflow of the digital signal decomposition algorithm to obtain the signal and noise components of extracellular and intracellular recording. **g** Examples of extracellular potential (top in blue) and intracellular potential (bottom in red) recorded using NWMEA before (left) and after (right) electroporation. The extracellular and intracellular potential recordings were decomposed into signal and noise components

After electrical characterization, we packaged the NWMEA into a NWMEA-based device to record the electrophysiological activity of cardiomyocytes in vitro. The NWMEA was assembled on an adaptive printed circuit board with a size of 2 cm × 2 cm and was covered with a PDMS chamber for cell culture (Fig. 3a). HL-1 cardiomyocytes were cultured on the device to test the biocompatibility of the NWMEA, focusing on whether the nanowell structures on electrodes prevented cell growth. After over 72 h of culture, a bright-field microscope was used to observe the growth and proliferation of HL-1 cardiomyocytes on the NWMEA surface. As shown in Fig. 3b, c, HL-1 cardiomyocytes attached and spread well on the NWMEA. We further employed immunofluorescence staining with DAPI and Cy3 dyes to check the health of the cardiomyocytes. As shown in Fig. 3d, DAPI-labeled nuclei (blue color) and Cy3-labeled α-actinin (red color) were clearly visualized inside individual cardiomyocytes. The well-defined actinin filaments demonstrated a healthy environment for the growth of cardiomyocytes. All the results above suggested that the NWMEA-based device had good biocompatibility and allowed healthy growth of cardiomyocytes for further electrophysiology study.

To test the electrophysiological recording capacity of NWMEA, the NWMEA-based device worked with a specified instrument to record the extracellular and intracellular potentials of HL-1 cardiomyocytes. Cellular electroporation by NWMEA applied a series of biphasic pulses to generate a nanowell electrical field and perforated cardiomyocyte membrane, which enabled microelectrodes to access the cytoplasm of cardiomyocytes through nanowells and thus recorded potential changes inside the cell membrane as intracellular recordings (Fig. 3e). The extracellular and intracellular recordings were decomposed into target signal and noise data from the environment by a customized algorithm (Fig. 3f). A typical sample in Fig. 3g shows that the extracellular recording had a small cellular potential signal with high background noise from the environment, and the noise almost outstripped the potential signal. The poor quality recording exhibited a low SNR for electrophysiological study. To improve the SNR of the NWMEA recording, we changed the recording from extracellular mode to intracellular mode by cellular electroporation, which enhanced cell membrane permeability and electrical coupling at the cell-electrode interface. Contrary to the extracellular recording, the intracellular recording showed a high potential signal and low background noise against the environment (Fig. 3g). In this typical sample, the intracellular signal was 2 times the extracellular signal, and the noise of intracellular recording was 40% of the noise of extracellular recording. The SNR of the intracellular recording was more than 4 times that of extracellular recording.

### Signal-to-noise analysis of extracellular and intracellular recordings using the NWMEA

**Fig. 4:**
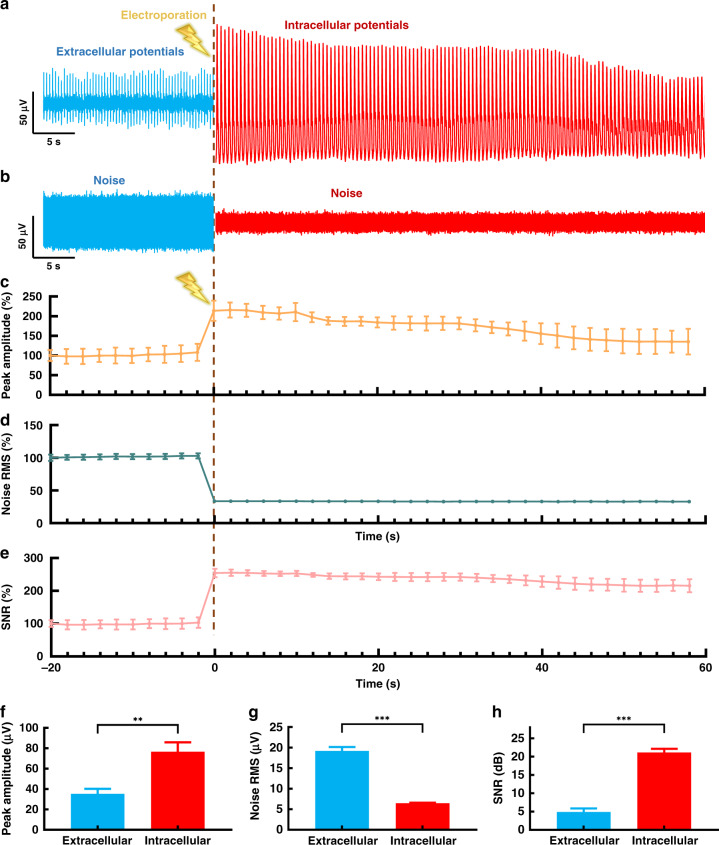
Performance comparison of the extracellular and intracellular potential recordings of cardiomyocytes using the NWMEA device. Performance comparison of the extracellular and intracellular potential recordings of cardiomyocytes using the NWMEA device. **a** The signal and **b** noise components decomposed from the extracellular and intracellular potential signals before and after NWMEA electroporation. Normalized statistical changes in **c** peak amplitude, **d** noise RMS and **e** SNR of the extracellular and intracellular potential signals before and after electroporation by the NWMEA. The statistical changes were normalized as 100% at 20 seconds before electroporation, and the time interval was 2 seconds. The data were obtained by electroporation in six channels. Statistical comparisons of **f** peak amplitude, **g** noise RMS and **h** SNR of the extracellular and intracellular potentials before and after electroporation. *n* = 6, *p < 0.05, **p < 0.01, and ***p < 0.001 in the unpaired *t* test

The datasets of long-term intracellular and extracellular recording were analyzed by the effective decomposition algorithm to demonstrate the improvement in the signal quality of the electrophysiological recording by electroporation with the NWMEA. The decomposition algorithm provided target signal and noise data of an instantaneous electrophysiological recording of HL-1 cardiomyocytes before and after NWMEA electroporation (Fig. 4a, b, respectively). The signals showed twenty seconds of extracellular recording on the left of a crimson dashed line, which indicated the application of electroporation through the NWMEA. Another sixty seconds of intracellular recordings to the right of the crimson dashed line are also presented for comparison. Before the electroporation, the recording of HL-1 cardiomyocytes presented low spike amplitudes of approximately 50 μV with significant noise even after digital signal processing (Fig. 4a, b). Upon electroporation via the NWMEA, the spike amplitudes immediately increased over 100 μV due to the nanoscale penetration of the cell membrane by the ‘electric nanoscissors’. The spike amplitude after electroporation by the NWMEA increased by a factor of 2, while the noise amplitude dropped more than 50% from ~50 μV to ~20 μV.

To statistically analyze the dynamic change in the NWMEA recording with electroporation, we divided the signal trace into short pieces with a two-second time interval for statistical analysis and used the normalized peak amplitude, noise root-mean-square (RMS) and signal-to-noise ratio (SNR) as metrics to evaluate the signal quality. Figure 4c–e shows the dynamic change in the signals before and after NWMEA electroporation. Normalized peak amplitudes of the recording were maintained at approximately 100% before electroporation and surged to approximately 220% after electroporation. The normalized amplitudes returned to approximately 130% after sixty seconds due to gradual resealing of the local cellular membrane (Fig. 4c). In contrast to the peak amplitude, the RMS, a metric indicating noise level, was initially approximately 100% and decreased to approximately 33% after electroporation (Fig. 4d). The signal quality was also described by the SNR, which increased from 100% to 254% and fell back to approximately 210% after electroporation (Fig. 4e). Both RMS and SNR plots showed a recovery to the initial state over time after electroporation. The analysis of normalized amplitude, RMS and SNR in the dynamic trend demonstrated that the NWMEA manipulating and recording significantly improved the signal quality of electrophysiological recording by achieving intracellular recording. The highest quality signals with the largest amplitude increase appeared at the moment after the electroporation by the NWMEA.

To further quantify the improvement in signal quality by the NWMEA, we compared the statistical results of 20 seconds of the recording data before electroporation (−20–0 s) and another 20 s of the recording data after electroporation (0–20 s). As shown in Fig. 4f, the peak amplitude of the extracellular recording was 66.79 ± 13.33 μV, while the amplitude of the intracellular recording after electroporation was 114.48 ± 23.13 μV, which was approximately twice that of the extracellular recording. The noise RMS of the extracellular recording was 20.01 ± 0.83 μV, while it was 6.53 ± 0.36 μV after electroporation. The noise RMS of the intracellular recording dropped to approximately 0.33 times that of the extracellular recording (Fig. 4g). Due to the higher signal amplitude and lower noise, the intracellular recording had a high SNR of 24.68 ± 2.02 dB, which was approximately 4 times that of the extracellular recording (Fig. 4h). The SNR of the intracellular recording was also higher than that of the conventional MEA recording and much closer to the SNR reported by patch clamp in electrophysiological recording^37,38^.

### Comparison between NWMEA and NPMEA recordings using time-domain analysis

**Fig. 5:**
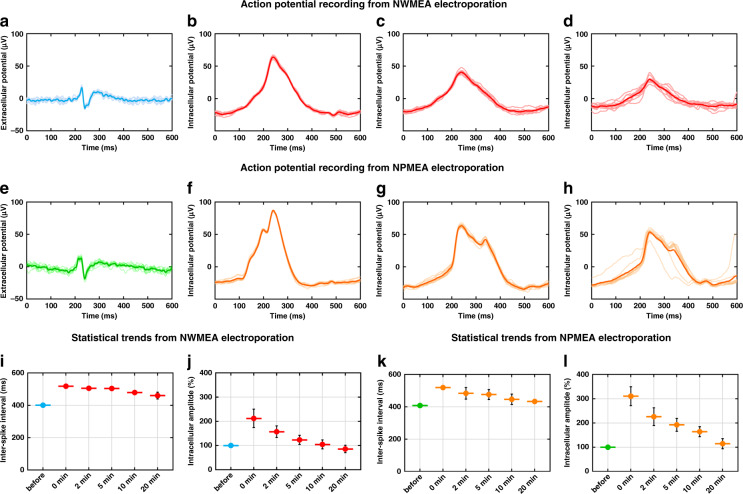
Performance comparison of the NWMEA and NPMEA devices in recording the action potentials of cardiomyocytes. (**a**–**c**) Action potential recording of cardiomyocytes using the NWMEA device before and after electroporation. Single action potentials were extracted from the recordings **a** before electroporation, **b** at the moment of electroporation, **c** 2 min after electroporation, and **d** 5 min after electroporation. **e**–**h** Action potential recording of cardiomyocytes using the NPMEA device before and after electroporation. A single action potential was extracted from the recordings **e** before electroporation, **f** at the moment of electroporation, **g** 2 min after electroporation, and **h** 5 min after electroporation. **i**–**j** Statistical trends of (**i**) interspike interval and (**j**) intracellular potential amplitude extracted from the NWMEA recordings at different time points. Statistical trends of (**k**) interspike interval and (**L**) intracellular amplitude extracted from the NPMEA recordings at different time points. In the graphs of the action potentials, the individual action potentials were averaged over 20 seconds after the selected time points. Solid and transparent lines denote the averages of the action potentials and individual action potentials, respectively. *n* = 6, *p < 0.05, **p < 0.01, ***p < 0.001 in the unpaired *t* test

After demonstrating the SNR improvement in the NWMEA recording, we further examined the efficacy of the NWMEA in recording intracellular action potentials by a comparison with a previously reported NPMEA, whose fabrication was more difficult than that of the NWMEA. The preparation of planar electrodes for the NPMEA is similar to the fabrication of the NWMEA. Planar electrodes were fabricated by successively depositing a Cr layer and Au layer on a silicon wafer. The Au pads were passivated by SiO_2_ layers, and a focused ion beam was applied to mill holes through passivation. Compared to the fabrication process of the NWMEA, one more step was required for the fabrication of the NPMEA; that is, Pt was deposited in the nanosized wells. Individual potentials were extracted from the electrophysiological data of cardiomyocytes measured by the NWMEA and NPMEA and compared in the time domain to study the capacities of the NWMEA and NPMEA for intracellular potential capture (Fig. 5a–h). The waveforms of potentials recorded by the NWMEA and NPMEA before the electroporation were similar to small pulse signals that always appeared in the extracellular potential recording by the conventional planar MEA. After electroporation, the waveforms of potentials included several profiles, such as rising section, falling section, and refractory periods, whose characteristics were consistent with the intracellular action potentials in previous works^39,40^. Similar to capture by patch clamp, the NWMEA and NPMEA both successfully transformed the individual recorded potentials from shapes of pulse signals into shapes of action potentials. We further compared the recordings of the NWMEA and NPMEA after electroporation and found that the potential amplitudes of the NWMEA were lower than those of the NPMEA after electroporation (Fig. 5b–d, f–h). The intracellular potential measured by the NWMEA dropped from ~90 μV to ~46 μV, and the intracellular potential measured by the NPMEA decreased from ~116 μV to ~84 μV 5 min after electroporation (Fig. S1).

To compare time-dependent changes in the NWMEA and NPMEA recordings with statistical analysis, we quantified the time-domain characteristics of the recordings with several time intervals in long-term recordings. The interspike interval and intracellular amplitude of the action potential both showed significant differences between the recordings before and after electroporation for these two nanostructures. The interspike intervals of the NWMEA and NPMEA recordings both increased from approximately 400 ms to more than 500 ms (401.16 ± 0.04 ms to 517.91 ± 0.62 ms for NWMEA and 408.45 ± 6.80 ms to 518.80 ± 0.24 ms for NPMEA) (Fig. 5i– k). After electroporation, the interspike intervals gradually returned to the initial values before electroporation and finally remained approximately 10% larger than the extracellular values. The potential amplitude of the NWMEA and NPMEA recordings showed the same trend, with a dramatic increase and a slow recovery within 20 min after electroporation (Fig. 5j, l). The same change in the interspike intervals and potential amplitude implied that compared to the NPMEA, electroporation by the NWMEA efficiently achieved recording of the intracellular potential and had a great capacity for long-term recording.

### Comparison between NWMEA and NPMEA recordings using nonlinear dynamic analysis

**Fig. 6:**
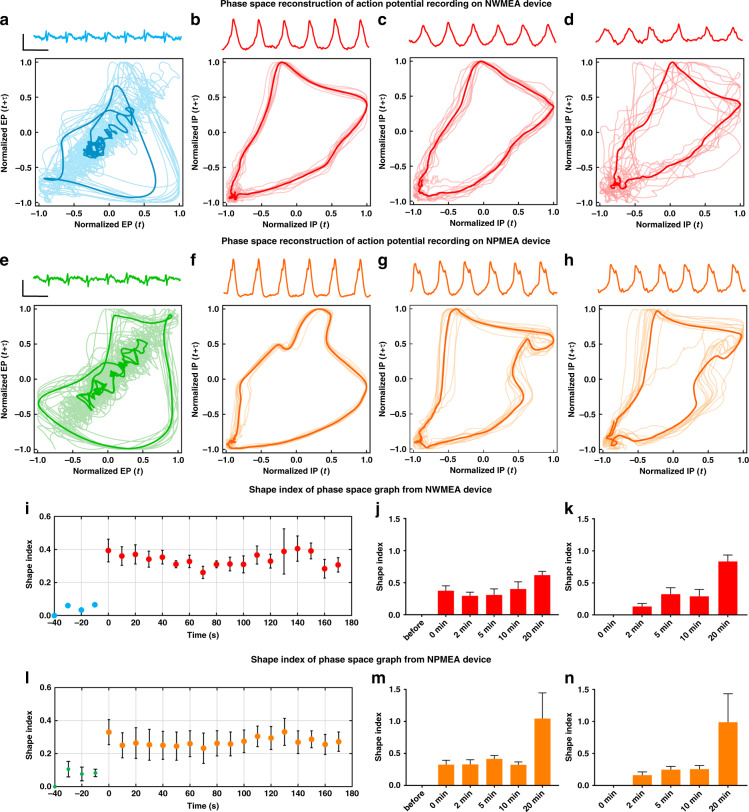
Nonlinear dynamic analysis of the action potential recording of cardiomyocytes using NWMEA and NPMEA devices. (**a**-**d**) Two-dimensional phase space reconstruction of the action potential recordings of cardiomyocytes using the NWMEA device before and after electroporation. The graphs were reconstructed **a** before electroporation, **b** at the moment of electroporation, **c** 2 min after electroporation, and **d** 5 min after electroporation. **e**–**h** Two-dimensional phase space reconstruction of the action potential recordings of cardiomyocytes using the NPMEA device before and after electroporation. The graphs were reconstructed **e** before electroporation, **f** at the moment of electroporation, **g** 2 min after electroporation, and **h** 5 min after electroporation. **i** The short-term shape indices for the NWMEA biodevice immediately after electroporation compared to those before electroporation. **j** The long-term shape index variations after electroporation compared to those before electroporation for the NWMEA. **k** The long-term shape index variations after electroporation compared to those immediately after electroporation for the NWMEA. **l** The short-term shape indices for the NPMEA biodevice immediately after electroporation compared to those before electroporation. **m** The long-term shape index variations after electroporation compared to those before electroporation for the NPMEA. **n** The long-term shape index variations after electroporation compared to those immediately after the electroporation NPMEA. In the reconstructed graphs, the contour plots were averaged over 20 s after the selected time points. Solid and transparent lines denote the averages of the contour plots and contour plots for individual action potentials, respectively. *n* = 6, *p < 0.05, **p < 0.01, and ***p < 0.001 in the unpaired *t* test

Considering the similar efficacy of the NWMEA and NPMEA recordings in conventional time-domain analysis, we further converted the NWMEA and NPMEA recordings from the time domain into two-dimensional phase space by nonlinear dynamic analysis to study the differences. We first compared the two-dimensional phase space reconstruction of the NWMEA and NPMEA in each trace at different time points before and after electroporation. The results from NWMEA and NPMEA both changed their reconstructed plots after electroporation due to the shift from extracellular to intracellular potential recording (Fig. 6a–h). After electroporation, the reconstructed plots also changed with time because the resealing of the cardiomyocyte membrane decreased the quality of the recording. These results were consistent with the time-domain analysis for the NWMEA and NPMEA recordings. Then, the reconstructed plots of the NWMEA and NPMEA were compared at different time points of their recordings. The graph difference between the NWMEA and NPMEA recordings in the phase space was more prominent than that in the time domain.

To further explore the difference between the NWMEA and NPMEA recordings, the differences in the time-dependent two-dimensional phase space graphs of the recordings were quantified. We first focused on the graph difference in phase space with the time around electroporation. The graph difference in the NWMEA and NPMEA recordings was studied by comparing the recordings of 170-s duration after electroporation with the recordings from 40-second duration before the electroporation, which served as the control. The quantification showed that the graph difference in the NWMEA and NPMEA recordings both increased from 0 to 0.1 before electroporation and went into a range of 0.2–0.4 after electroporation (Fig. 6i, l). The averaged graph shape index of the NWMEA increased approximately 8 times after electroporation, while the averaged graph shape index of NPMEA increased only approximately 4 times after electroporation. This implied the higher efficiency of the NWMEA-based device to switch the extracellular potential recording into intracellular potential recording.

The shape index changes in the phase space reconstruction with time in long-term recordings were also quantified. The recordings before the electroporation were used as the control to indicate the long-term shape index change in the phase space graph in the whole recording (Fig. 6j, m). The shape indices of the NWMEA and NPMEA remained in the range of 0.3~0.4 from 0 min to 10 min after electroporation. However, the average of the shape indices from NPMEA measurements increased dramatically to ~1.0, and the average of the shape indices from NWMEA measurements increased to ~0.6 at 20 min after electroporation. The shape indices compared to the value at 0 min showed a similar increase at 20 min after electroporation for both the NWMEA and NPMEA (Fig. 6k, n). The increases suggested that the measurement environment of the NWMEA and NPMEA changed significantly between 10 min and 20 min after electroporation and turned into poor-quality recordings. This drastic change between 10 min and 20 min after electroporation was only observed in nonlinear dynamic analysis with the two-dimensional phase space reconstruction but not in the time-domain analysis of the NWMEA and NPMEA recordings. The NPMEA recordings showed large variances in the recordings of different microelectrodes. The statistical error bar of the NPMEA recording was ~7 times that of the NWMEA, which implied that the NWMEA could provide a more repeatable recording, especially when the recordings became poor quality due to cardiomyocyte membrane resealing.

## Discussion

Our work presented an NWMEA-based biosensing system for cellular electroporation to record the intracellular potential of cardiomyocytes. The biosensing system successfully combined NWMEA fabrication, bioelectronic execution, and a data analysis algorithm to achieve a high-quality recording of intracellular action potentials from a single cell. First, the NWMEA devices were successfully fabricated by drilling micron-sized nanowell arrays on a conventional planar MEA. The nanowell structure on the surface of MEA generated a nanoscale electrical field when applied with driving voltage and penetrated the cardiomyocyte membrane for intracellular potential recordings. The microscale of nanowell arrays guaranteed a single cardiomyocyte recording of action potential. Second, the bioelectronic execution used a waveform generator and voltage amplifier system to perform cellular electroporation and electrophysiological measurements, respectively. The bioelectronic execution enabled the NWMEA to penetrate the cell membrane and record the intracellular potential. Third, the potential recordings were decomposed into signal and noise components by the digital wavelet transforms in the data analysis algorithm. Taking advantage of these three parts, the SNR of the intracellular recording by the NWMEA was approximately 4 times that of the extracellular recording, whose electrophysiological recording was much closer to the recording of conventional MEA recordings^22,41,42^. During electroporation, the intracellular action potential amplitude gradually transitioned back to extracellular features, which could be attributed to membrane resealing after electroporation^43,44^ (Fig. 5J). We also observed no significant changes in the frequency and amplitude compared to the values of extracellular recording. This may, to some extent, demonstrate the health condition of the cells after electroporation. Hence, secondary electroporation could be carried out by the NWMEA to further confirm the healthy state of the cells after the first electroporation in our future work. Overall, our work demonstrated that the NWMEA device successfully acquired high-quality intracellular potential recordings from cultured cardiomyocytes with minor damage to cells.

It has been reported in previous work that one more step to fabricate the nanopillars after obtaining the nanowells was required for the NPMEA structure^45^. This process requires high alignment precision and stability. Four limitations can be concluded: (1) NPMEA requires more fabrication processes. (2) In situ deposition with high precision is time-consuming. (3) The nanopillars possibly exhibit imperfect contact between the pillar-planar electrodes and lower the success rate of NPMEA fabrication. (4) The deposited nanopillars generally need posttreatment to improve the conductivity. All these factors may affect the post electroporation consistency. In contrast, our NWMEA device extracted the qualified intracellular potentials with reduced fabrication processes (Figs. 5, 6), although the NWMEA obtained a relatively lower amplitude than the NPMEA in the intracellular potential recordings of cardiomyocytes. The nonlinear dynamic analysis in phase space showed that the shape indices of the NWMEA and NPMEA recordings changed slightly within 10 min after electroporation. Nevertheless, they both increased significantly approximately 20 min after electroporation. This suggested that the time between 10 min and 20 min after electroporation was the main attenuation stage of intracellular recordings by the NWMEA and NPMEA. This phenomenon might be induced by the resealing of the cardiomyocyte membrane^43,44^. Then, we compared the shape indices of the NWMEA and NPMEA recordings at 20 min after electroporation. The mean and variance in the shape index from NPMEA recordings were both larger than those from NWMEA recordings. This suggested that the NWMEA provided more accurate and stable recordings of intracellular potentials in a long-term recording. In summary, although the NWMEA acquired a lower amplitude of intracellular action potential than the NPMEA, the NWMEA is able to measure more stable intracellular potentials in a long-term recording with reduced complexity in device fabrication when compared to the NPMEA.

Beyond studies with NWMEA and NPMEA, our work can also contribute to the strategy of the design and development of other nanostructure microelectrode devices. The nanowell is a downward structure on the microelectrode, which is different from commonly reported upgrowing nanostructures such as nanopillars, nanomushrooms and nanovolcanos. Nanowell structures can penetrate the cell membrane and acquire high-quality intracellular signals as well as upgrowing nanostructures. Meanwhile, downward nanowells can be more easily fabricated than upgrowing nanostructures. Thus, our novel nanofabrication strategies that changed upgrowing fabrications into downward fabrications can expand the search space for other nanostructured microelectrode fabrication processes. These strategies would thus benefit electrophysiological sensors that require large-scale manufacturing.

## Conclusions

In summary, we presented a biosensing system incorporating an NWMEA to record intracellular action potentials from a single cardiomyocyte. The biosensing system successfully assessed the intracellular potential of cardiomyocytes by nanowell electroporation and measured the intracellular action potential, with a higher SNR than the extracellular potential. The NWMEA is easier to fabricate than other MEA devices with upgrowing nanostructures, and it enables the biosensing system to record intracellular potential with a remarkable SNR. The NWMEA-based biosensing system can provide an alternative tool to record the intracellular action potential of a single cell and broaden the usage of MEAs in electrophysiological investigations.

## Supplementary information

Supplemental Material
