# Supplementary material for: A biosensing system employing nanowell microelectrode arrays to record the intracellular potential of a single cardiomyocyte
Source: Microsyst Nanoeng. 2022 Jun 27;8:70. doi: 10.1038/s41378-022-00408-9 (PMC9237042; doi:10.1038/s41378-022-00408-9)
Supplement: Supplementary file 1 — Supplemental Material [file 41378_2022_408_MOESM1_ESM.docx]

**Supplementary Information**


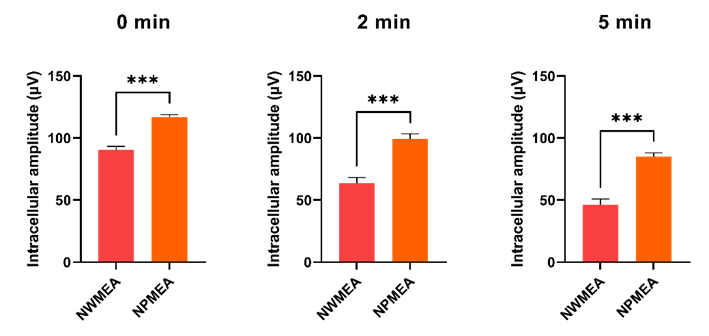


Figure S1. Statistical comparisons of peak amplitudes of NWMEA and NPMEA intracellular recordings at 0 min, 2 min an 5 min. n=10, * p < 0.05, ** p < 0.01, *** p < 0.001 in unpaired t-test.
